# Retrospective Analysis of 118 Patients With Cutaneous T-Cell Lymphomas: A Single-Center Experience

**DOI:** 10.3389/fonc.2022.884091

**Published:** 2022-06-07

**Authors:** Kamila Polgárová, Jindřich Polívka, Ondřej Kodet, Pavel Klener, Marek Trněný

**Affiliations:** ^1^ First Dept. of Internal Medicine - Hematology, University General Hospital in Prague and First Faculty of Medicine, Charles University, Prague, Czechia; ^2^ Institute of Hematology and Blood Transfusion, Prague, Czechia; ^3^ Department of Dermatovenerology, University General Hospital in Prague and First Faculty of Medicine, Charles University, Prague, Czechia; ^4^ Institute of Pathological Physiology, First Faculty of Medicine, Charles University, Prague, Czechia

**Keywords:** cutaneous T-cell lymphoma (CTCL), mycosis fungoides (MF), sézary syndrome (SS), real-world analysis, retrospective study

## Abstract

Cutaneous T-cell lymphomas (CTCL) represent rare non-Hodgkin lymphomas (NHL) with an incidence less than 1 per 100,000 inhabitants. The most common type of CTCL is mycosis fungoides (MF), which represents approximately 60% of all CTCL, followed by Sézary syndrome (SS), approximately 5%. We retrospectively analyzed the outcome of 118 patients with MF (n=96) and SS (n=22) treated between the years 1998 and 2021 at the Charles University General Hospital in Prague, Czech Republic. The ratio between men and women was 1.2:1 (62 men, and 56 women). The median age at diagnosis was 62 years (23 to 92 years). From the MF cohort 48 patients (50% out of MF cohort) presented with advanced stage disease. Ninety patients (77%) received a systemic treatment at any time from the diagnosis; the median number of therapy lines was two. At the time of database lock, the overall survival (OS) of 96 patients with MF reached 17.7 years with the median follow-up 4.0 years. With the median follow-up 2.6 years, the median OS of 22 patients with SS was 3.5 years. The most common type of systemic therapy for MF included low-dose methotrexate (61%), interferon-alpha (58%), bexarotene (28%), and chlorambucil (25%). The most common type of therapy for SS included bexarotene (64%), extracorporeal photopheresis (50%), and interferon-alpha (45%). Only the minority of patients received innovative targeted agents including brentuximab vedotin, mogamulizumab, or pembrolizumab. Besides the retrospective analysis of the CTCL cohort, current standards and future perspectives of selected innovative agents are summarized and discussed. The analyzed cohort represents the largest cohort of CTCL patients in the Czech Republic. Overall, the survival parameters of our CTCL cohort are comparable to those previously published by other groups. In conclusion, our analysis of 118 real world cohort of consecutive CTCL patients treated at the single center confirmed the efficacy of immune response modifiers and underlines the urgent need for ample implementation of innovative agents and their combinations into earlier lines of therapy.

## Introduction

Cutaneous T-cell lymphomas (CTCL) are a heterogeneous group of non-Hodgkin lymphomas (NHL) that present primarily in the skin and have no evidence of extracutaneous involvement at the time of diagnosis, but can progress to systemic disease (1). CTCL constitutes approximately 4% of all NHL, with an incidence of around 0.8/100,000 person-years. The prevalence of CTCL is up to ten times higher. In the Czech Republic, the incidence of CTCL was 0.4 cases per 100,000 population in 2015 (2). Mycosis fungoides (MF) and Sézary syndrome (SS) are the most common subtypes of CTCL, representing almost 70% of all CTCL (MF 62%, SS 3-5%) (3, 4).

TNMB (tumor, node, metastasis, blood) staging still plays an important role in the decision making for each patient’s therapeutic strategy (5). To date, several prognostic indexes have been established. For early-stage MF, the cutaneous lymphoma international prognostic index (CLIPi) is calculated based on male sex, age > 60 years, presence of plaques, folliculotropism, and lymph node stage N1/Nx. The similar CLIPi for advanced stage MF/SS includes male sex, age > 60 years, lymph node stage N2/N3, blood stage B1/B2, and visceral involvement (stage M1) (6). Most recently, the Cutaneous Lymphoma International Consortium (CLIC) prognostic index for advanced MF/SS was proposed based on four independent adverse factors: age > 60 years, large cell transformation (LCT), stage IV, and elevated lactate dehydrogenase (LDH) (7). However, published reports validating their use have yielded conflicting results (8, 9).

MF and SS remain incurable malignancies with chronic and relapsing clinical course, and OS of approximately 18 years (5, 7, 10) and 3 years respectively (11). Patients with early-stage MF are usually treated with skin-directed therapy (SDT) only. The first-line systemic therapy of advanced-stage CTCL or SS is still based on immune response modifiers, including interferon-alpha (IFNα), low-dose methotrexate (LD-MTX), or bexarotene (12–14); these can also be used as a maintenance therapy after total skin electron beam therapy (15). Other treatment options for relapsed or refractory (R/R) CTCL patients include histone deacetylase inhibitors (HDACi, romidepsin, vorinostat), conventional and newly formulated cytostatics (e.g. gemcitabine, platin derivatives, liposomal doxorubicin), antibody-drug conjugates (brentuximab vedotin, BV), check-point inhibitors (pembrolizumab), or glycoengineered monoclonal antibodies mogamulizumab (MOGA) (16). Allogeneic hematopoietic stem cell transplantation (HSCT) should be considered for eligible patients as a potentially curative therapy in selected cases (12–14, 17).

HDACi (vorinostat, romidepsin) were incorporated into the treatment of MF in the first decade of the new millennium. The pivotal trials lead to their approval for CTCL patients by The United-States Food and Drug Administration (FDA) and Chinese and Japanese authorities, but not by the European Medicine Agency (EMA). Additional data from clinical trials, as well as real-world evidence of HDACi efficacy remain conflicting with overall response rates (ORR) 6-40% and time to next treatment (TTNT) of 3-4 months (18, 19). On the other hand, BV, an anti-CD30 Ab-drug conjugate, showed convincingly superior results with ORR of 55% in R/R CTCL and MF patients when compared to bexarotene or LD-MTX in the phase III ALCANZA trial and was approved by both, FDA and EMA (20–22). Similarly, MOGA, a monoclonal antibody targeting C-C chemokine receptor type 4 (CCR4), showed significantly better responses when compared to vorinostat and was approved by FDA and EMA (23–26). Pembrolizumab is currently not approved by the authorities for the treatment of CTCL patients; however, a recent trial (NCT02243579) showed its efficacy in R/R advanced MF and SS (16).

Despite the recent approval of several innovative targeted agents, many questions remain to be answered including the optimal sequence of immune response modifiers and different innovative agents or finding the most effective rational drug combinations (27, 28).

Here, we present a single-center retrospective analysis of 118 patients with CTCL treated at the Charles University General Hospital for last 23 years. Besides the retrospective analysis of the CTCL cohort we summarize and discuss efficacies, side effects, accessibility, and future perspectives of selected innovative agents.

## Patients and Methods

### Patients

Data were collected from 118 consecutive patients with biopsy-proven diagnoses of MF and SS treated at the Charles University General Hospital in Prague between the years 1998 – 2021. The study was approved by the University General Hospital Ethics Committee (number 1816/15 S-IV).

The diagnosis of MF and SS was established or revised according to the 2018 WHO-EORTC classification (1). Collected data included the patient demographics, clinical and pathologic findings including the disease stage with TNMB classification of MF and SS that was proposed and revised in 2007 by the International Society for Cutaneous Lymphomas (ISCL) and by the European Organization of Research and Treatment of Cancer (EORTC) (29, 30). Different clinic pathological variants, as well as the history of large cell transformation (LCT) were also evaluated. Risk stratification using CLIPi and CLIC prognostic indexes was performed as well. In this study, a systemic therapy was defined as an oral or intravenous chemotherapy, immunotherapy, course of immune response modifiers, or extracorporeal photopheresis (ECP). Polychemotherapy regimen administered to at least one patient included: COP (cyclophosphamide, vincristine, prednisone), CHOP (cyclophosphamide, vincristine, doxorubicin, prednisone), CHOEP (cyclophosphamide, vincristine, doxorubicin, etoposide, prednisone), ICE (ifosfamide, carboplatin, etoposide), and CMED (cyclophosphamide, methotrexate, etoposide, dexamethasone).

### Statistical Analysis

OS was calculated from the date of diagnosis to the patient’s death or the date of the last follow-up. OS for the whole cohort was estimated using the Kaplan-Meier method. Comparison between curves and univariate analysis of factors of possible prognostic significance was made by the log-rank test and Cox proportional hazard regression analysis. P values below 0.05 were considered significant. After examining each variable separately by univariate analysis, a multivariate model using a backward-stepwise approach was performed to select variables with the most predictive power (p<0.25).

## Results

### Baseline Characteristics of Analyzed Patients

A total of 118 consecutive patients with CTCL (MF and SS) referred to our tertiary hematologic center were included in our analysis. Staging categories and prognostic factors frequency of the analyzed patients are provided in **Table 1**. Briefly, median age at diagnosis was 62 years (23–92) with men to women ratio 1.2:1 (62 men [52.5%], 56 women [47.5%]). Ninety-six (81.4%) patients were diagnosed with MF, and 22 (18.6%) patients with SS. Median time from the development of the first symptoms to the diagnosis of MF and SS were 3.9 and 2.4 years, respectively. Out of the 96 MF patients, 48 patients (50%) had advance-stage disease (≥ IIB), and 48 the early-stage disease at the time of diagnosis. Folliculotropic MF variant was observed in 7 patients (5.9%), while neither pagetoid reticulosis nor granulomatous slack skin were detected. At the time of dataset closure, 79 patients were alive. Lactate dehydrogenase (LDH) > upper limit of normal (ULN) was observed in 49 patients, most of whom (n=39) had advanced-stage disease.

**Table 1:** Summary of demographic, clinical staging characteristics, prognostic factors, and OS of the analyzed patients.

Parameter	number of patients	% of the whole cohort	median OS (95% CI)(years)
whole cohort	118	100.0	17.7
age in Dg ≤ 60 years	52	44.1	not reached
age in Dg > 60 years	66	55.9	5.3 (3.3-7.3)
women	56	47.5	17.7 (1.1-34.3)
men	62	52.5	not reached
Mycosis fungoides	96	81.4	not reached
- Early stage (<IIB)	48	40.7	not reached
- Advanced stage (≥IIB)	48	40.7	5.9 (2.9-8.8)
Sézary syndrome	22	18.6	3.5 (2.2-4.9)
Staging			
IA	32	27.1	not reached
IB	10	8.5	not reached
IIA	6	5.1	not reached
IIB	33	28.0	7.6 (4.1-11.1)
IIIA	8	6.8	2.1 (0.5-3.7)
IIIB	5	4.2	4.5 (-1.5-10.5)
IVA1	21	17.8	3.2 (1.8-4.7)
IVA2	3	2.5	2.1 (0.7-3.5)
T1	35	29.7	not reached
T2	14	11.9	not reached
T3	37	31.4	7.6 (-3.9-19.1)
T4	32	27.1	2.8 (1.5-4.1)
Nx	39	33.1	4.2 (3.0-5.5)
N0-N1	73	61.8	not reached
N2	3	2.5	5.1 (5.1-5.1)
N3	3	2.5	2.2 (0.5-3.9)
B0	86	72.9	Not reached
B1	7	5.9	1.2 (1.0-1.4)
B2	22	18.6	3.5 (2.3-4.8)
M0	118	100.0	17.7
≥ 10 000 Sézary cells/µl	9	7.6	2.6 (-0.9-6.1)
Folliculotropic variant	7	5.9	not reached

Dg, diagnosis; LCT, large cell transformation; ULN, upper limit normal. Staging: T1-T2 – patches and plaques covering <10% or ≥10% of skin surface; T3 – skin tumors. T4 – erythrodermia (covering ≥80% body surface area). N0 – no clinically abnormal lymph nodes (LN); Nx clinically abnormal LN, without histologic confirmation; N1 – clinically abnormal LN, histopathology Dutch grade 1; N2 clinically abnormal LN, histopathology Dutch grade 2; N3 – clinically abnormal LN, histopathology Dutch grade 3-4; M0 – no visceral organ involvement; B0 – no significant blood involvement (≤5% Sézary cells from lymphocytes); B1 – low blood tumor burden (Sézary cells >5% of peripheral blood lymphocytes, but not meeting criteria for B2); B2 – high blood tumor burden (≥1000/µl Sézary cells with positive clone).

### Prognostic Indexes

According to CLIPi, 23, 17, and 8 patients with early-stage disease fulfilled the criteria for low-risk, intermediate-risk, and high-risk disease, respectively. Twenty-nine, 27, and 14 patients with advanced-disease fulfilled the criteria for low-risk, intermediate-risk, and high-risk categories, respectively. According to CLIC prognostic index for advanced stage CTCL, 32 patients had low-risk disease, 15 intermediate-risk, and 23 had high-risk disease (**Supplementary Table 1**).

### Large Cell Transformation and Organ Involvement

Large cell transformation (LCT) was diagnosed in 16 patients (13.6%), from which 7 had advanced stage disease. The median time from the diagnosis of MF or SS to LCT was 1.2 years (0-16.8). Four patients were diagnosed with LCT at the time of diagnosis; the rest was diagnosed during the disease course. None of our patients had confirmed organ involvement at the time of diagnosis. However, three patients developed organ involvement during the disease course - one patient with central nervous system involvement (with massive cerebrospinal fluid and parenchymal infiltration), one with gastrointestinal tract involvement and one with respiratory tract involvement,. All these patients had poorly controlled disease; two patients died of progressive diseases, while the only survivor is currently planned for HSCT.

### Skin Directed Therapy (SDT)

**Figure 1:**
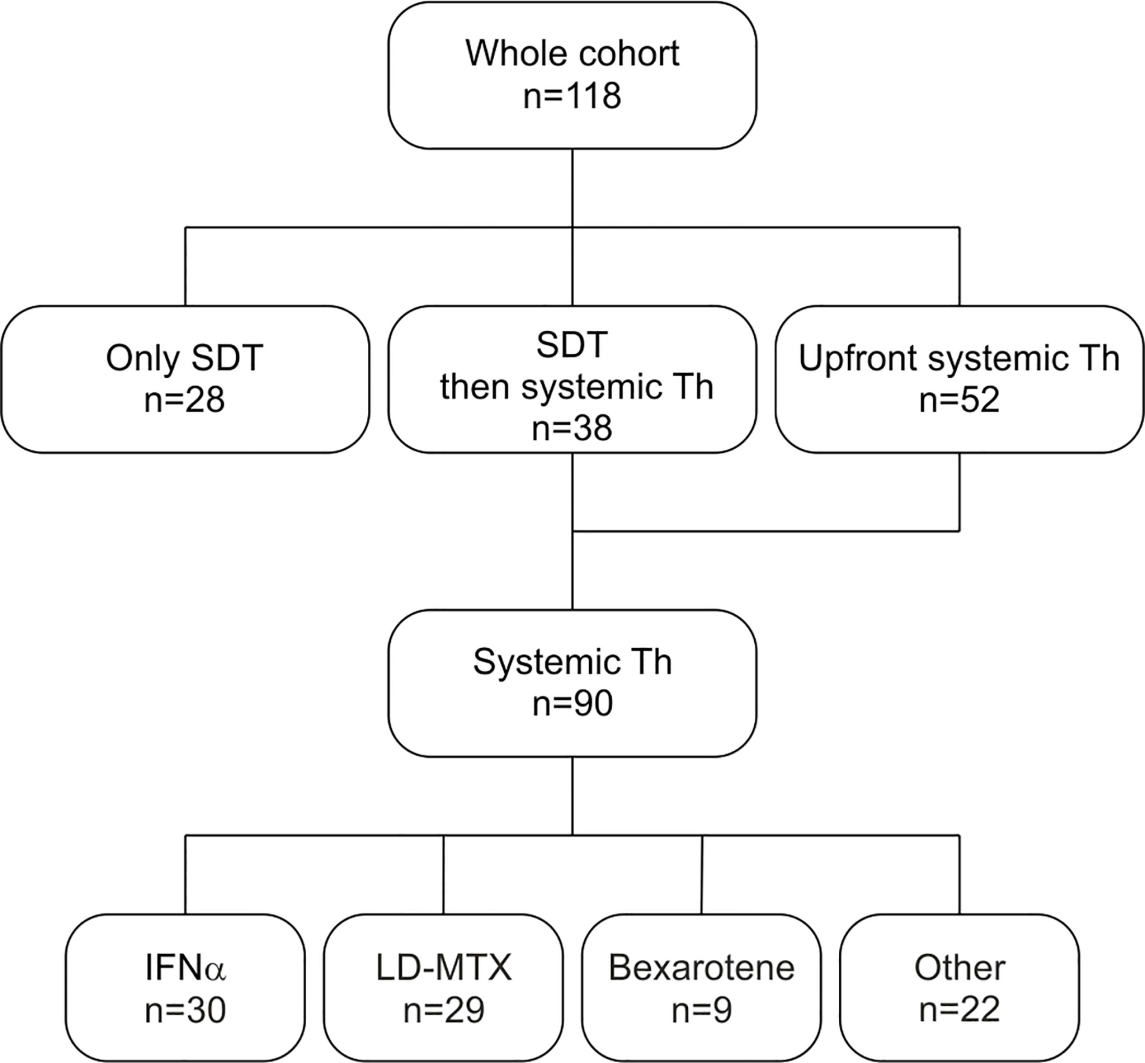
Flowchart of administrated therapies – SDT and 1^st^ line systemic therapy. Other systemic agents include IFNα+LD-MTX, chlorambucil, single-agent- and polychemotherapy, systemic corticosteroids and hydroxyurea. SDT – skin directed therapy. Th – therapy. IFNα- interferon alpha. LD-MTX- low-dose methotrexate.

A flowchart of administered therapies is displayed in **Figure 1**. SDT (1 or 2 lines) preceded the use of systemic agents in 38 of these patients (31 and 7 patients, respectively). Twenty-eight (23.7%) patients were treated only by SDT, mostly by phototherapy (11 patients with UVB, 2 patients with UVA), PUVA (3 patients), localized radiotherapy (1 patient), local corticoids (used as single treatment in 9 patients), local retinoids (1 patient) and local imiquimod (1 patient). Of these patients, 3 were defined as advanced stage disease due to T3 cutaneous stage. Total skin electron irradiation (TSEI) was used in 19 patients (16.1%), mostly as third- and fourth-line treatment with total cumulative doses ranging between 6 to 52 Gy (median 36 Gy), and was always followed by systemic consolidation, usually by INFα, LD-MTX or their combination, 2 patients received maintenance by bexaroten.

### Systemic Therapy

Systemic therapy (with or without SDT) was used in 90 patients (77.1%), 68 with MF (70.8% from the MF cohort), and 22 with SS (100%). The median time from the diagnosis to the systemic therapy was 3.4 months (0-268 months). The median number of systemic treatment lines for patients with MF and SS was 2 (1–10) and 4 (1–11), respectively.

The first line of systemic therapy was based predominantly on IFNα (30 patients), LD-MTX (29 patients), and their combinations. Bexarotene and chlorambucil were used in nine and seven patients, respectively (**Table 2**). Single agent cytostatics or polychemotherapy regimens were rarely used in the upfront setting. For SS the first line therapy was based mainly on IFNα.

**Table 2:** Systemic agents used as first-line treatment for patients with MF and SS with or without previous skin directed therapy.

First-line systemic therapy	total	MF	SS	≥ IIB stage
IFNα	30	23	7	14
LD-MTX	29	27	2	15
Bexarotene	9	7	2	2
Chlorambucil	7	4	3	3
IFNα + LD-MTX	3	2	1	2
polychemotherapy	5	4	1	4
Single-agent chemotherapy	3	1	2	1
Systemic corticosteroids	3	0	3	0
Hydroxyurea	1	0	1	0

Single-agent chemotherapy included gemcitabine, cyclophosphamide and etoposide. Polychemotherapy included COP, CHOP and CHOEP. INFα, interferon-alpha; LD-MTX, low-dose methotrexate.

Immune response modifiers (IFNα, LD-MTX, and bexarotene) alone or in combination were the most frequently used systemic agents in the 1^st^ to 3^rd^ systemic therapy line (**Table 3**). Thirty-three patients (24 MF [19 ≥IIB], 9 SS) were treated with single agent chlorambucil, which was mostly used either as a bridge for other therapy or as palliative treatment. Single-agent chemotherapy (gemcitabine, cyclophosphamide, etoposide, intermediate-dose MTX) was used in 17 patients with advanced-stage disease (12 MF, 5 SS). Polychemotherapy (COP, CHOP, CHOEP, ICE, or CMED regimen) were used in 15 patients, all with advanced-stage disease (11 MF, 4 SS). Other strategies included extracorporeal photopheresis (ECP), used in twelve patients as third and subsequent line therapy, and alemtuzumab used in seven patients as fifth and subsequent line of therapy. HDACi were not used as they are not approved by EMA or by local authorities.

**Table 3:** Systemic agents used in all treatment lines in whole cohort and numbers of patients treated by them in particular clinical groups.

Systemic agents used in all therapeutic lines	total	MF	SS	≥ IIB stage	mostly used as
IFNα	66	56	10	32	1^st^-2^nd^ line
LD-MTX	66	59	7	33	1^st^-3^rd^ line
Bexarotene	41	27	14	17	3^rd^ line
Chlorambucil	33	24	9	19	2^nd^-3^rd^ line
Single agent chemotherapy	17	12	5	12	within all lines
Polychemotherapy	15	11	4	11	within all lines
ECP	12	1	11	1	≥ 2^nd^ line
Alemtuzumab	7	2	5	2	≥4^th^ line
Brentuximab-vedotin	5	3	2	2	≥5^th^ line
Pembrolizumab	1	0	1	0	9^th^ line
Mogamulizumab	2	1	1	1	≥10^th^ line

Single agent chemotherapy included gemcitabine, cyclophosphamide, etoposide, and intermediate dose methotrexate (500mg/m^2^). Polychemotherapy included COP, CHOP, CHOEP, ICE and CMED. Several patients were treated by more than one line of single agent or polychemotherapy. IFNα, interferon-alpha; LD-MTX, low dose methotrexate; ECP, extracorporeal photopheresis.

Innovative targeted agents including BV, MOGA, and pembrolizumab were used in five, two and one patient, respectively, after more than five previous lines of systemic therapy. Two patients were treated by more than one of these agents sequentially.

Two patients underwent autologous SCT (ASCT) as consolidation (both MF). One patient underwent ASCT for relapsed Hodgkin lymphoma without receiving systemic treatment for MF. One patient with SS underwent allogeneic HSCT with alemtuzumab as a bridge therapy.

### Response Assessment

Median TTNT for the patients who received systemic therapy of any kind was 6.4 months. Median TTNT in the cohort of patients treated with IFNα, LD-MTX, and bexarotene was 7.3 months (1.5-170.8), 9 months (1.4-55.4), and 6.8 months (1.2-58), respectively. Median TTNT in the cohort patients treated with the combination of IFNα and LD-MTX was 7.6 months (0.8-51.5); median TTNT for patients treated with combination of IFNα plus bexarotene was 5.3 months (2.1-51.0).

Notably, median TTNT in the cohort of patients with erythrodermic MF and SS (16 patients) treated with the triple combination of IFNα, bexarotene, and ECP reached 15.1 months (4.1-46.3). Median TTNT of patients treated by TSEI followed by systemic therapy was 7 months (0.5-16.6).

Single-agent chemotherapy, as well as polychemotherapy led only to limited response duration with median TTNT of 2.5 months (0.2-22) – **Supplementary Table 2**.

### Survival and Risk Factors

**Figure 2:**
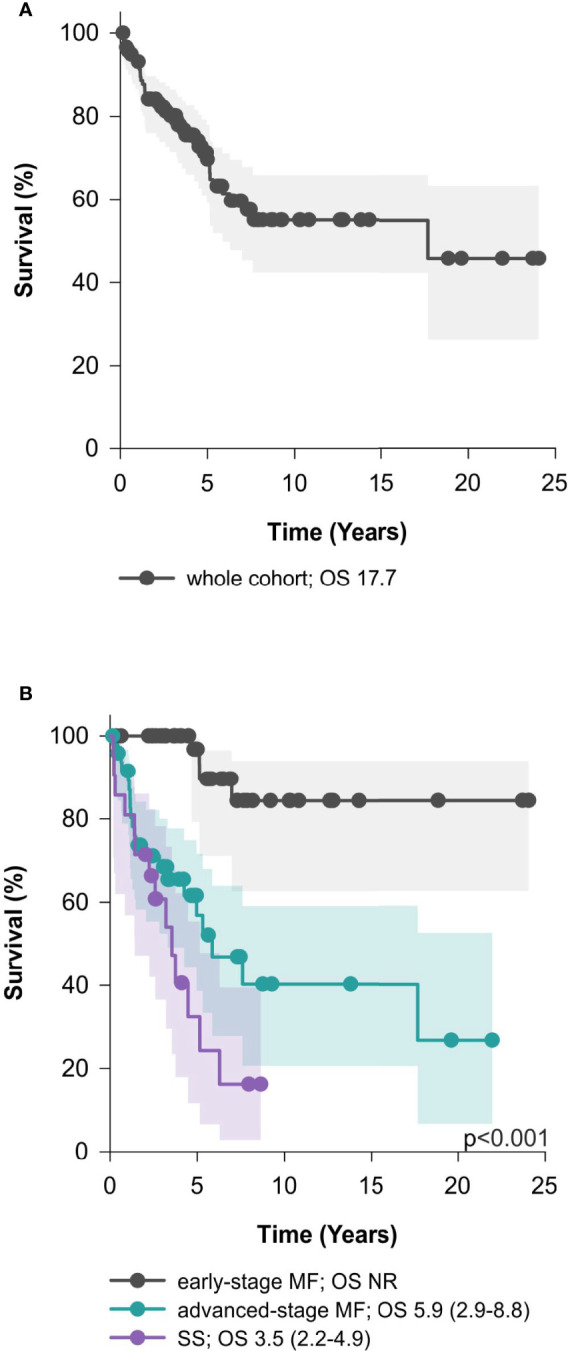
Survival parameters of the whole cohort – median overall survival (95% confidence interval). **(A)** Median Overall survival of the whole cohort; **(B)** median overall survival of early-stage MF, advanced-stage MF, and SS. MF, mycosis fungoides; SS, Sézary syndrome; OS, median overall survival; NR, not reached.

The median OS of the whole cohort of 118 patients was 17.7 years with no significant differences between men and women (**Figure 2A**). The median OS for early-stage MF was not reached. The median OS for advanced-stage MF and SS were 5.9 years (95% CI, 2.9-8.8) and 3.6 years (95% CI, 2.2-4.9), respectively. (**Figure 2B**).

### Statistical Analysis

Univariate analyses confirmed that extended skin involvement (≥ T3), blood involvement (≥ B1 and SS cells ≥ 10 000/μl), age (> 60 years), and LDH (> upper limit of normal) negatively correlated with survival (**Supplementary Table 3 and Supplementary Figure 1**). On the other hand, a correlation of LCT or folliculotropic variant with survival was not observed. Multivariate analysis confirmed T4 stage and nodal involvement as independent prognostic factors for the whole cohort as well as for advanced stages.

Using clinical prognostic factors CLIC index we stratified patients with advanced stage MF into low, intermediate, and high-risk groups. Only patients with advanced-stage disease (but not early-stage disease) were stratified according to CLIPi into low, intermediate, and high-risk groups (**Supplementary Figure 2**).

**Figure 3:**
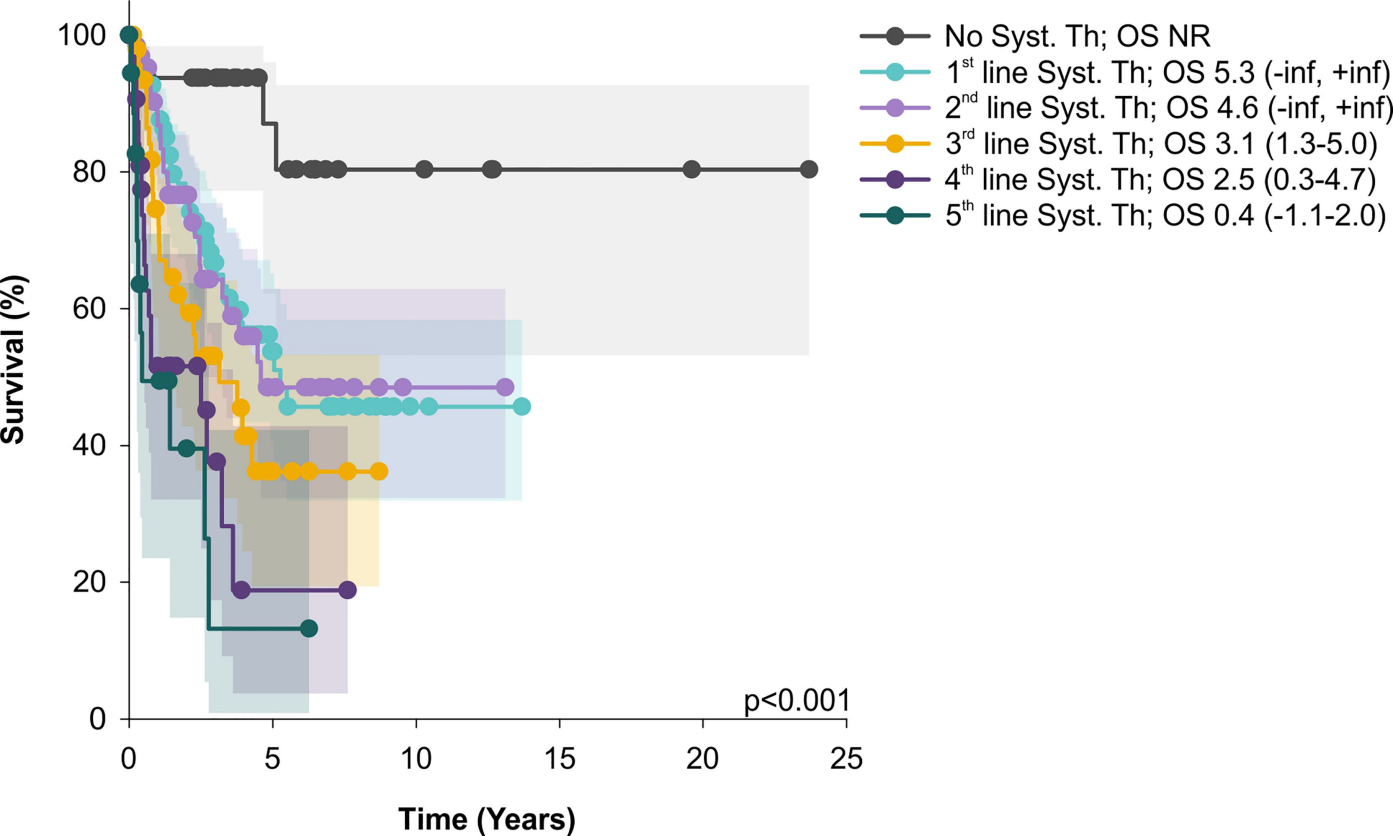
OS of patients treated only by skin directed therapy significantly differs from those with systemic treatment (Syst. Th); similarly, OS of patients treated by 1 and 2 lines of systemic therapy was significantly better than OS of patients after 4 or 5 systemic treatment lines. Median overall survival and 95% CI are showed. OS, median overall survival; CI, confidence interval; NR, not reached.

From other factors, a total number of systemic therapy lines negatively correlated with survival (**Figure 3**).

### Other Malignancies

Other malignancies (before or after the diagnosis of CTCL) were observed in twenty patients (16.9%), most frequently hematologic malignancies (ten patients) and five skin cancers (**Supplemental Table 4**).

## Discussion

In this study, we retrospectively analyzed a single-center cohort of 118 patients with MF and SS treated in our tertiary center. The OS of the whole cohort was 17.7 years, with median OS of the SS cohort 3.5 years, and expected differences between the OS of early-stage MF (median OS not reached) and advanced-stage MF (median OS 5.3 years). This is in line with the previously published data (31). Besides the stage, univariate analyses confirmed that age (> 60 years), LDH (> ULN), skin involvement (T3-T4), blood involvement (B1 and B2), and TNMB staging negatively correlated with survival. Concerning skin involvement, the median OS of T3 group (7.6 years) was longer than previously reported and in contrast to the so far published data, differences in OS between T3 and T4 involvement was observed (32–34).. On the other hand, we did not observe a significant difference in OS between B1 and B2 blood involvement, probably due to the low numbers of patients in the B1 group. Interestingly, we confirmed the adverse prognostic impact of Sézary cell counts as reported by Alberti-Violetti et al. with median OS of 2.6 years in the subcohort of patients with ≥ 10 000/µl compared to 6.3 years for the patients with less than 10 000 Sézary cells/µl (33). Folliculotropic variant or LCT did not correlate with survival, again probably due to low numbers of cases. We did not observe any difference in OS between men and women, either, in contrast to reports of worse survival in men (5). Concerning the prognostic indices, patients with advanced stage were stratified into separate prognostic subgroups according to both CLIPi and CLIC prognostic index (6, 7). However, we did not observe any significant difference in survival between the intermediate and high-risk groups (in both scoring systems). Moreover, our data did not validate the prognostic power of CLIPi in patients with early stage disease. This is probably due to underrepresentation and selection bias of patients with early stage disease in our cohort, since these patients were often treated by a secondary care specialist solely and not referred to our tertiary center. Even though improving health-related quality of life (HRQoL) is an important aim for patients with MF/SS (35, 36), data comparing pre- and post-treatment HRQoL were not available in our retrospective cohort due to frequent referrals from other centers and long study interval. The use of HRQoL questionnaires is, however being implemented in our center.

Ninety patients from our cohort received systemic therapy, most frequently the immune response modifiers. In the MF cohorts, the most often used first-line treatment was IFNα followed by LD-MTX. In the SS cohort, the most often used first-line treatment was also based on immune response modifiers, e.g. IFNα followed by ECP and/or combination with bexarotene.

A rapid shortening of OS observed with increasing lines of systemic therapies suggests that implementation of innovative agents is requisite to improving the outcome of R/R MF/SS patients. Also, conventional chemotherapy, often used in advanced treatment settings, leads only to short lived disease control. Even though studies comparing chemotherapy with biological agents are lacking, our data confirm the already observed inability of chemotherapeutic agents to provide durable responses (37).

In our study, only five patients were treated with new drugs, not before 5 previous systemic lines of therapy, all during year 2020 and 2021. The main reason for the observed administration of innovative agents exclusively in the heavily pretreated patients was the low rate of both BV and MOGA reimbursements by the health insurance companies (**Supplemental Figure 3**).

Dozens of new promising anti-cancer molecules are currently being evaluated in numerous clinical trials in patients with CTCL. These comprise diverse immunotherapy molecules, e.g. lacutamab- a monoclonal antibody against KIR3DL2 (NCT03902184), AFM13- a bispecific antibody anti-CD30 x anti-CD16A (NCT04101331), SAR442257- a trispecific antibody anti-CD38 x anti-CD28 x anti-CD3 (NCT04401020), CAR T-lymphocytes (NCT04712864) (16, 38, 39), or CAR NK cells (NCT03081910). Several small molecular weight inhibitors were reported active in CTCL patients including HDAC inhibitor panobinostat (NCT01261247) (40), PI3K inhibitor duvelisib (41), immunomodulatory agent lenalidomide (42) or proteasome inhibitor ixazomib (43). Besides single-agent approaches, rational combinations currently being tested in clinical trials comprise BV plus lenalidomide (NCT03302728, NCT03409432), pembrolizumab plus gemcitabine (NCT04960618), duvelisib plus nivolumab (NCT04652960) or romidepsin (44). Also, novel markers predicting efficacy of the targeted treatment are currently being evaluated (45–47).

Despite recent clinical approvals of several innovative targeted agents and ongoing clinical testing of new molecules with promising activity, many questions remain to be answered. These include optimal sequencing of immune response modifiers and different innovative agents or finding the most effective drug combinations (27, 28).

In conclusion, our analysis of real-world cohort of 118 consecutive CTCL patients treated at our center confirmed good efficacy of immune response modifiers and underlines the urgent need for broad implementation of innovative agents and their combinations into earlier lines of therapy.

## Data Availability Statement

The original contributions presented in the study are included in the article/**Supplementary Material**. Further inquiries can be directed to the corresponding author.

## Ethics Statement

The studies involving human participants were reviewed and approved by Ethics Committee of the General University Hospital, Prague, Approval Number 1816/15 S-IV. Written informed consent for participation was not required for this study in accordance with the national legislation and the institutional requirements.

## Author Contributions

KP analysed the data, wrote the manuscript, and prepared most Figures and Tables. JP evaluated the data and contributed to the interpretation of the results and manuscript preparation. OK and PK contributed to result interpretation and manuscript revision. MT contributed to interpretation of the data and revised the paper. All authors contributed to the article and approved the submitted version.

## Funding

The study was supported by Ministry of Health of the Czech Republic grant AZV NV19-08-00144, Charles University Center of Excellence UNCE/MED/016, and National Institute for Cancer Research (ID LX22NPO5102) Funded by the European Union - Next Generation EU, Programme EXCELES.

## Conflict of Interest

The authors declare that the research was conducted in the absence of any commercial or financial relationships that could be construed as a potential conflict of interest.

## Publisher’s Note

All claims expressed in this article are solely those of the authors and do not necessarily represent those of their affiliated organizations, or those of the publisher, the editors and the reviewers. Any product that may be evaluated in this article, or claim that may be made by its manufacturer, is not guaranteed or endorsed by the publisher.
