# Supplementary material for: Retrospective Analysis of 118 Patients With Cutaneous T-Cell Lymphomas: A Single-Center Experience
Source: Front Oncol. 2022 Jun 7;12:884091. doi: 10.3389/fonc.2022.884091 (PMC9210166; doi:10.3389/fonc.2022.884091)
Supplement: Supplementary file 1 [file DataSheet_1.docx]

Supplementary Material

**Retrospective analysis of 118 patients with cutaneous T-cell lymphomas: a single-center experience**

Kamila Polgárová^1†^, Jindřich Polívka^2†^, Ondřej Kodet^3^, Pavel Klener^1,4*^, Marek Trněný^1^

^1^First Dept. of Internal Medicine - Hematology, University General Hospital in Prague and First Faculty of Medicine, Charles University, Prague, Czech Republic

^2^Institute of Hematology and Blood Transfusion, Prague, Czech Republic

^3^Department of Dermatovenerology, University General Hospital in Prague and First Faculty of Medicine, Charles University, Prague, Czech Republic

^4^Institute of Pathological Physiology, First Faculty of Medicine, Charles University, Prague, Czech Republic

^†^ These authors share first authorship

**Supplementary Table 1.** Summary of prognostic scores’ categories, their frequency and their association with overall survival (OS). CI – confidence interval.

| **CLIPi** | number of patients | % of the whole cohort | median OS (95% CI)  (years) |
| --- | --- | --- | --- |
| early low,  early intermediate  early high | 48 | 40.7 | not reached |
| late low | 28 | 23.7 | 17.7 (3.0-32.4) |
| late intermediate | 28 | 23.7 | 3.7 (2.6-4.9) |
| late high | 14 | 11.9 | 2.2 (0.5-3.9) |
| **CLIC prognostic index** |  |  |  |
| low | 32 | 27.1 | 7.6 (-4.1-19.3) |
| intermediate | 15 | 12.7 | 4.2 (2.9-5.6) |
| high | 23 | 19.5 | 3.2 (1.5-4.9) |

**Supplementary Table 2.** Time to next treatment (TTNT) for particular systemic approaches used alone or in combination. Agents used as clearly stated bridge therapy for other treatment were excluded. IFNα – interferon-alpha. LD-MTX – low dose methotrexate. ECP – extracorporeal photopheresis. TSEI – total skin electron irradiation.

| **Therapy** | TTNT, months (median) | TTNT, months (range) | number of evaluable patients |
| --- | --- | --- | --- |
| Chemotherapy | 2.5 | 0.2 - 22.0 | 31 |
| IFNα | 7.3 | 1.5 - 170.8 | 32 |
| LD-MTX | 9 | 1.4 - 55.4 | 26 |
| Bexarotene | 6.8 | 1.2 - 58.7 | 10 |
| IFNα + MTX | 7.6 | 0.8 - 51.5 | 21 |
| IFNα + bexarotene | 5.4 | 2.7 - 82.3 | 10 |
| IFNα + bexarotene + ECP | 15.1 | 4.1 - 46.3 | 8 |
| TSEI | 7 | 0.5 - 16.6 | 11 |
| alemtuzumab | 9.1 | 0.9 - 17.1 | 5 |

**Supplementary Table 3.** Summary of parameters evaluated in univariate analysis and their association with overall survival. HR – hazard ratio. CI – confidence interval. ULN – the upper limit of normal. MF – mycosis fungoides. SS – Sézary syndrome.

| **Covariate** | **HR (95% CI)** | **Overall P value** |
| --- | --- | --- |
| Sex (male vs. female) | 0.972 (0.516-1.829) | 0.92 |
| Folliculotropic variant | 0.385 (0.0528-2.815) | 0.27 |
| Large cell transformation | 0.587 (0.270-1.278) | 0.20 |
| LDH (≤ULN vs. ≥ ULN) | 3.289 (1.669-6.483) | <0.001 |
| Age (≤ 60 years vs. > 60 years) | 0.299 (0.144-0.618) | <0.001 |
| Clinical stage |  | <0.001 |
| Early-stage MF vs. late-stage MF | 0.123 (0.0419-0.359) | <0.001 |
| Late-stage MF vs. SS | 1.816 (0.916-3.600) | 0.09 |
| T stage |  | <0.001 |
| T1-2 vs. T3 | 0.173 (0.0563-0.533) | <0.001 |
| T3 vs. T4 | 3.352 (1.644 6.837) | <0.01 |
| N stage |  |  |
| N0 vs. Nx | 0.198 (0.0972-0.402) | <0.001 |
| N0 vs. N1 | 2.284 (0.294-17.747) | 0.43 |
| N0 vs. N2 | 8.620 (1.800-41.285) | 0.01 |
| N0 vs. N3 | 4.678 (1.033-21.197) | 0.05 |
| B stage |  | <0.001 |
| B0 vs. B1 | 5.626 (2.095-15.110) | <0.001 |
| B0 vs. B2 | 3.858 (1.907-7.808) | <0.001 |
| B1 vs. B2 | 0.736 (0.260-2.081) | 0.56 |
| SS cells in peripheral blood (≥ 10 000 vs. < 10 000/ µl) | 3.250 (1.072-9.850) | 0.03 |

**Supplementary Table 4.** Other malignancies diagnosed before, concurrently, or after MF and SS. Two patients had more than one cancer diagnosed besides MF/SS

| **Malignancy diagnosed before, concurrently, or after MF/SS** | **number of patients** |
| --- | --- |
| Skin malignancies | 5 |
| Spinocelullar carcinoma | 3 |
| Melanoma | 1 |
| Merkel cell carcinoma | 1 |
| Other malignancies | 7 |
| Ovarian carcinoma | 1 |
| Breast adenocarcinoma | 1 |
| Urothelial papillocarcinoma | 1 |
| Prostatic adenocarcinoma | 1 |
| Cervical carcinoma | 1 |
| Laryngeal carcinoma | 1 |
| Renal cell carcinoma | 1 |
| Hematologic malignancies | 10 |
| Mantle cell lymphoma | 2 |
| Hodgkin lymphoma | 2 |
| systemic peripheral T-cell lymphoma not otherwise specified | 1 |
| chronic lymphocytic leukemia | 1 |
| B-cell non-Hodgkin lymphoma not otherwise specified | 1 |
| Lymphomatoid papulosis | 1 |
| Myelodysplastic syndrome | 1 |
| JAK2+ myeloproliferative disease | 1 |


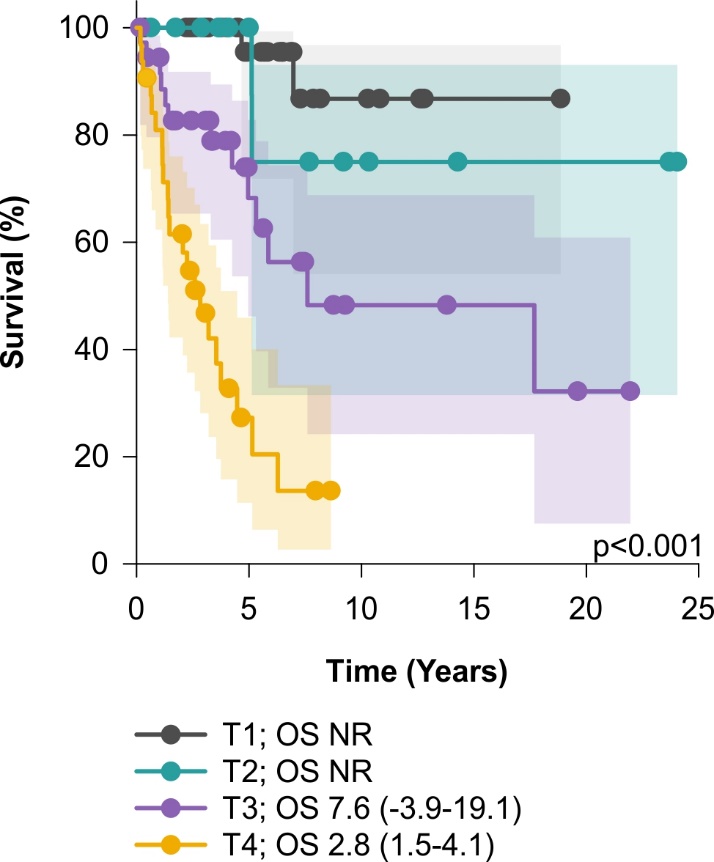


**A**


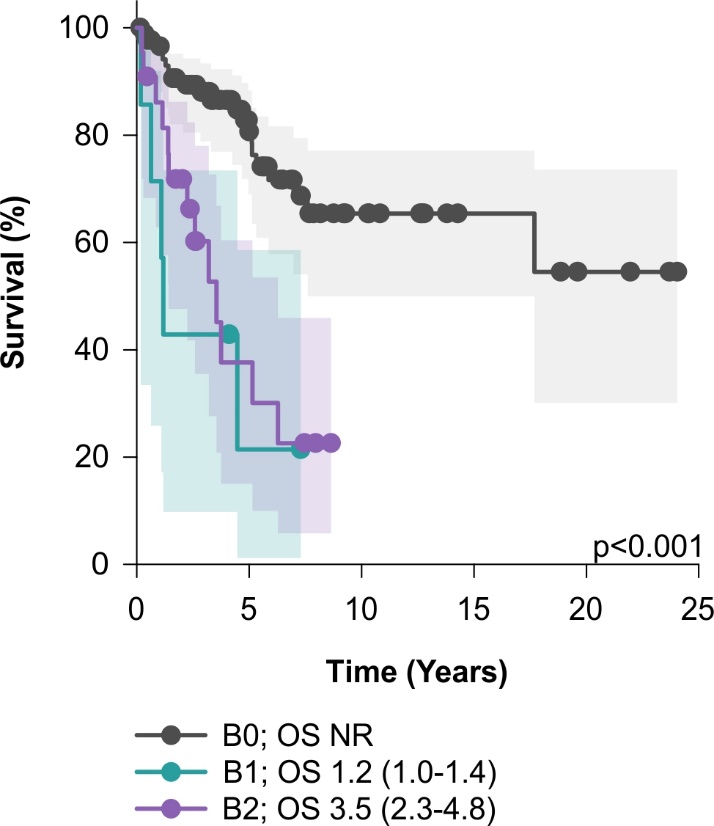


**B**


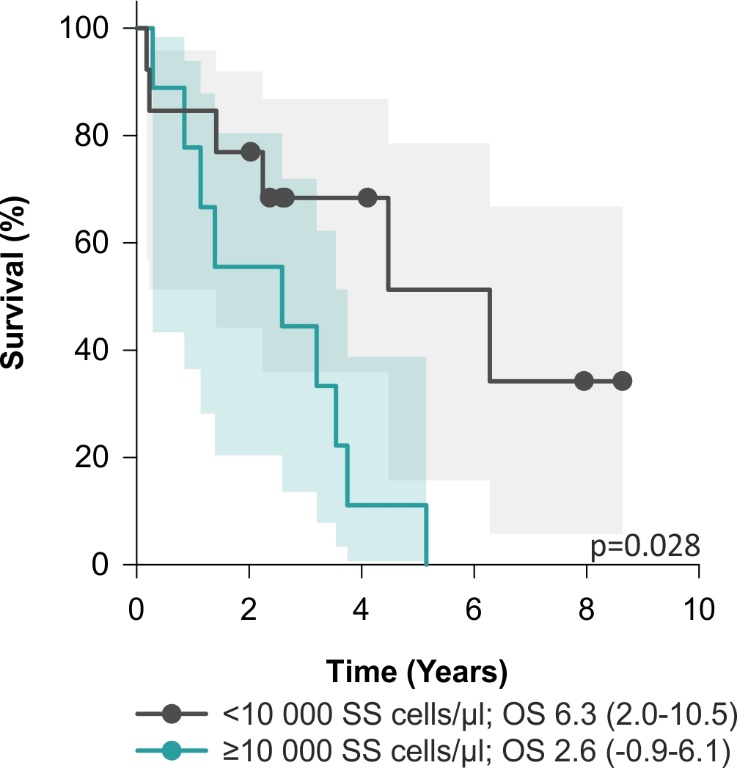


**C**


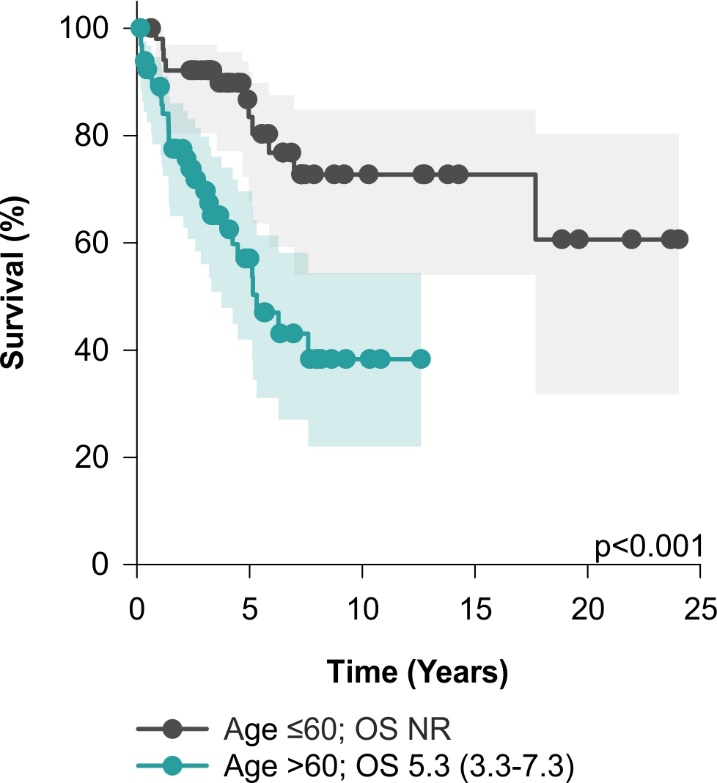


**D**


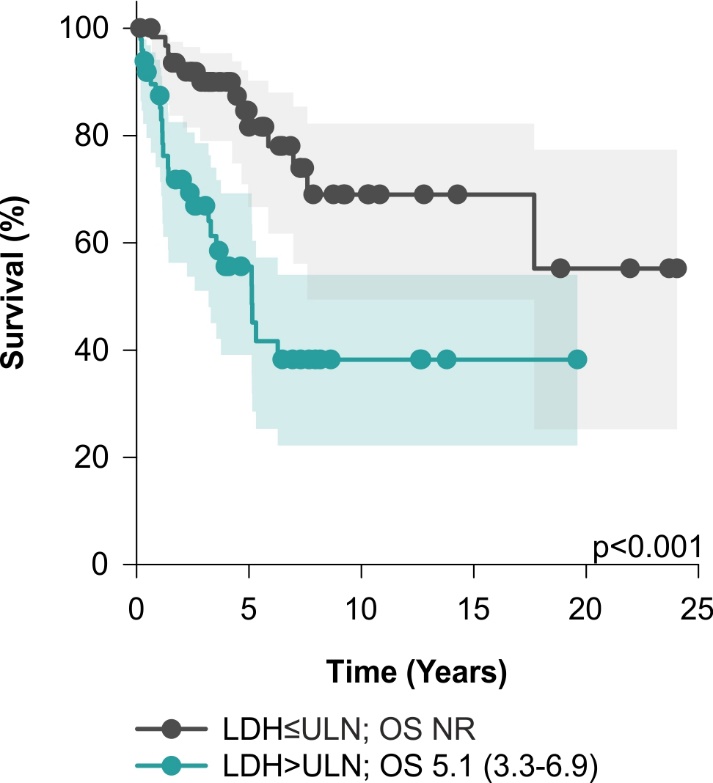


**E**

**Supplementary Figure 1.** Correlation of selected prognostic factors with overall survival in subcohorts of MF and SS patients: skin involvement (**1A**), blood involvement (**1B, 1C**), LDH (**1D**) and age (**1E**). OS – median overall survival. NR – not reached. MF – mycosis fungoides. SS – Sézary syndrome. ULN – the upper limit of normal.


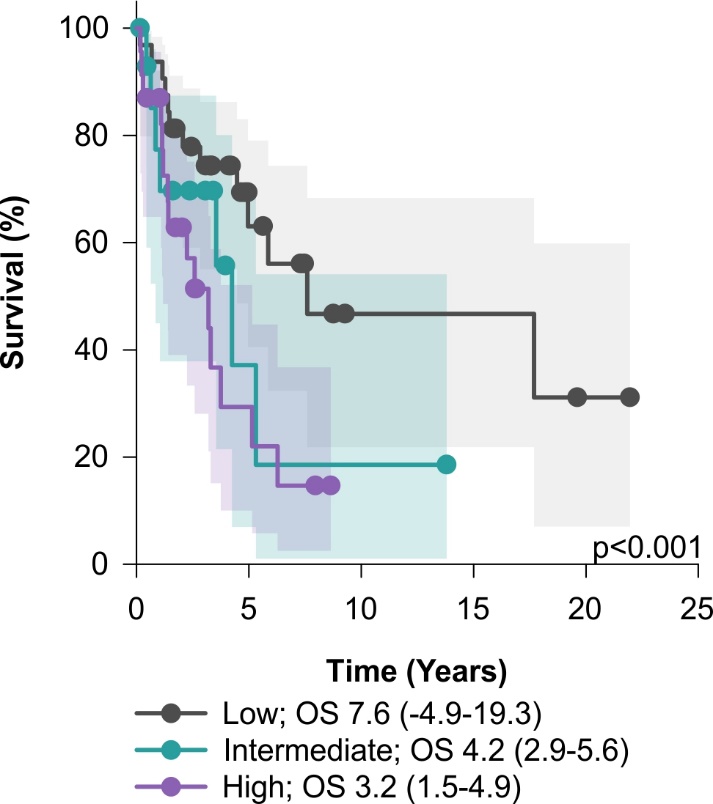


**A**


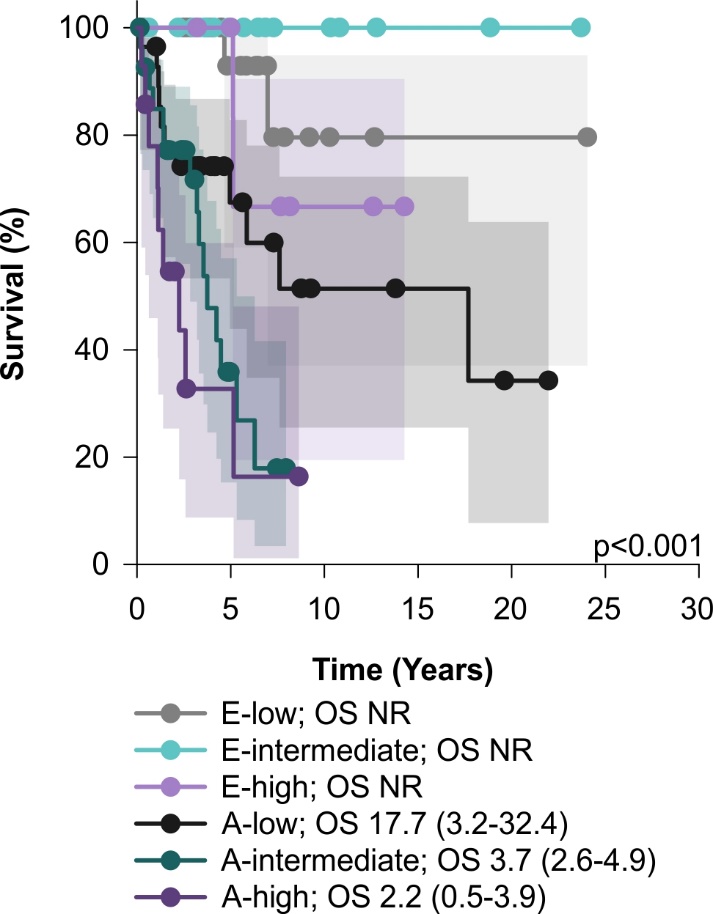


**B**

**Supplementary Figure 2.** Overall survival is stratified according to **CLIC** prognostic index used for advanced disease stages with low-risk patients having better OS than intermediate and high risk patients; no difference was observed between intermediate and high risk categories (**2A**). Overall survival of low, intermediate, and high risk **CLIPi** categories within early (E) and advanced (A) stage disease patients; in advanced stage disease the OS differed between low and the rest of risk categories. In early-stage disease the stratification did not reach significance (**2B**). OS – median overall survival. NR – not reached.


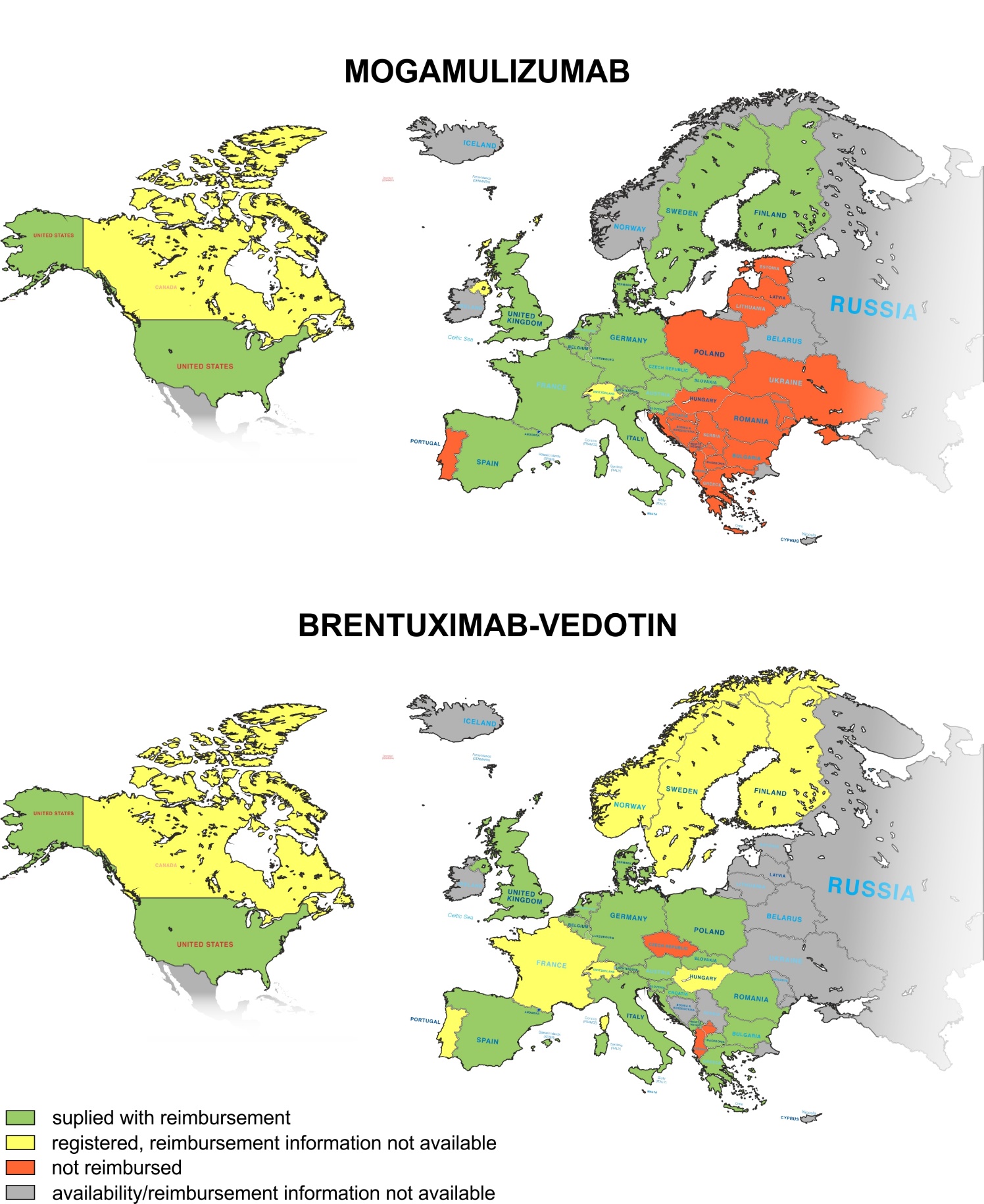


**Supplementary Figure 3.** Availability map showing supply and reimbursement of new drugs within North American and European countries as reported by producing company.
